# Survival of retinal ganglion cells after damage to the occipital lobe in humans is activity dependent

**DOI:** 10.1098/rspb.2018.2733

**Published:** 2019-02-27

**Authors:** Colleen L. Schneider, Emily K. Prentiss, Ania Busza, Kelly Matmati, Nabil Matmati, Zoë R. Williams, Bogachan Sahin, Bradford Z. Mahon

**Affiliations:** 1 Department of Brain and Cognitive Sciences, University of Rochester, Rochester, NY 14627, USA; 2 Medical Scientist Training Program, University of Rochester School of Medicine and Dentistry, Rochester, NY 14642, USA; 3 Department of Psychology, Carnegie Mellon University, Pittsburgh, PA 15206, USA; 4 Department of Neurology, University of Rochester Medical Center, Rochester, NY 14642, USA; 5 Department of Ophthalmology, University of Rochester Medical Center, Rochester, NY 14642, USA; 6 Department of Neurosurgery, University of Rochester Medical Center, Rochester, NY 14642, USA; 7 Department of Neurology, Rochester Regional Health, Rochester, NY 14621, USA; 8 Center for Visual Science, University of Rochester, Rochester, NY 14642, USA

**Keywords:** stroke, homonymous hemianopia, retinal ganglion cells, optical coherence tomography, functional magnetic resonance imaging, retinotopic mapping

## Abstract

Damage to the optic radiations or primary visual cortex leads to blindness in all or part of the contralesional visual field. Such damage disconnects the retina from its downstream targets and, over time, leads to trans-synaptic retrograde degeneration of retinal ganglion cells. To date, visual ability is the only predictor of retinal ganglion cell degeneration that has been investigated after geniculostriate damage. Given prior findings that some patients have preserved visual cortex activity for stimuli presented in their blind field, we tested whether that activity explains variability in retinal ganglion cell degeneration over and above visual ability. We prospectively studied 15 patients (four females, mean age = 63.7 years) with homonymous visual field defects secondary to stroke, 10 of whom were tested within the first two months after stroke. Each patient completed automated Humphrey visual field testing, retinotopic mapping with functional magnetic resonance imaging, and spectral-domain optical coherence tomography of the macula. There was a positive relation between ganglion cell complex (GCC) thickness in the blind field and early visual cortex activity for stimuli presented in the blind field. Furthermore, residual visual cortex activity for stimuli presented in the blind field soon after the stroke predicted the degree of retinal GCC thinning six months later. These findings indicate that retinal ganglion cell survival after ischaemic damage to the geniculostriate pathway is activity dependent.

## 1. Introduction

Damage to the optic radiations or primary visual cortex (V1) causes blindness in the contralesional visual hemifield of both eyes. Degeneration of ganglion cells in the retina has been detected following occipital lobe damage in post-mortem studies [1–3] and in *in vivo* studies [4–6] of monkeys, cats, and humans. Prior cross-sectional studies have shown that the distribution of retinal ganglion cell degeneration years after a stroke is spatially correlated with the persistent visual field defect [7–9].

One limitation of prior research relating retinal ganglion cell degeneration to visual field defects is that those studies do not take into account spontaneous visual recovery, which occurs to some degree in 50% of stroke patients with visual field cuts [10]. Therefore, it is important to separately determine the fate of ganglion cells in areas of the retina that correspond to regions of the visual field that recovered vision. In addition, the correlation between visual ability and retinal ganglion cell complex (GCC) thickness does not capture the potential role of the functionality of any remaining tissue in the visual cortex after the stroke. Prior functional magnetic resonance imaging (fMRI) studies of patients with homonymous visual field defects have demonstrated preserved V1 activity in response to stimulation of the phenomenal blind field [11–18]. Those findings suggest that blindness may not always be due to a lack of processing in V1, but perhaps to a combination of disordered processing in V1, de-efferentation of V1 from higher order visual areas, or other factors. Regardless of why some patients are blind despite V1 activity, it is clear that there can be a dissociation between phenomenal vision and V1 activity after injury to the geniculostriate pathway. In addition, a previous study found decreased lateral geniculate nucleus activation in patients with retrogeniculate lesions [19], suggesting alterations in the activity profile of neurons upstream of the lesion. These observations suggest an alternative approach for thinking about retinal ganglion cell atrophy post-stroke, namely that preservation of retinal ganglion cells is an activity-dependent process that is independent of, although correlated with, phenomenal vision.

Here, we test the hypothesis that the integrity of the GCC depends on stimulus-evoked activity in the early visual cortex. To test this hypothesis, we collected automated Humphrey perimetry, spectral-domain optical coherence tomography (OCT) of the macula and fMRI retinotopy data from stroke patients with visual field defects. All recruited patients received automated Humphrey perimetry as standard of care within the first two months post-stroke, allowing us to distinguish visual areas that were (i) unaffected by the stroke, (ii) initially blind and then recovered, and (iii) stably blind. We found that GCC thickness was reduced in stably blind areas, and, to a lesser extent, in recovered areas of the visual field. In patients tested greater than or equal to five months post-stroke, there was a positive relationship between visually evoked activity and GCC thickness that was specific to stably blind areas of the visual field. Ten of the 15 patients completed a study visit within two months of their stroke in addition to the visit five+ months after stroke (table 1; electronic supplementary material, figure S1). In these 10 patients, visual cortex activity at the first time point predicted subsequent GCC thinning in the region of the retina that corresponded to the original blind field.

**Table 1.** Participant demographics. RHH, right homonymous hemianopia; LHH, left homonymous hemianopia; RUQ, right upper quadrantanopia; RLQ, right lower quadrantanopia; LLQ, left lower quadrantanopia; U > L, upper quadrant greater than lower quadrant; # denotes patients in FLUORESCE trial (NCT02737930; see electronic supplementary material).

participant	age	gender	lesion	original field cut
1	55	M	right superiolateral parieto-occipital, ventral posteriomedial occipital	LHH
2	66	M	bilateral anteromedial occipital	LHH and RUQ
3	58	M	right medial occipital	LHH
4	59	F	right occipital	LHH
5	68	M	left thalamus and ventral occipital	RHH
6	57	F	left anteromedial occipital	RHH
7	72	F	right ventromedial occipital	incomplete LHH (U > L)
8	74	M	left parieto-occipital	RLQ
9	69	M	left temporal and ventral anteromedial occipital	RUQ
10	53	M	left temporo-occipital, right parieto-occipital	RUQ, incomplete LLQ
11^#^	43	M	left ventral medial occipital	incomplete RHH (U > L)
12^#^	71	M	left medial occipital	RHH
13^#^	70	M	right medial occipital	LHH
14^#^	61	M	right dorsal anteromedial occipital	LHH
15^#^	79	F	right lateral parieto-occipital	LHH

## 2. Results

**Figure 1.**
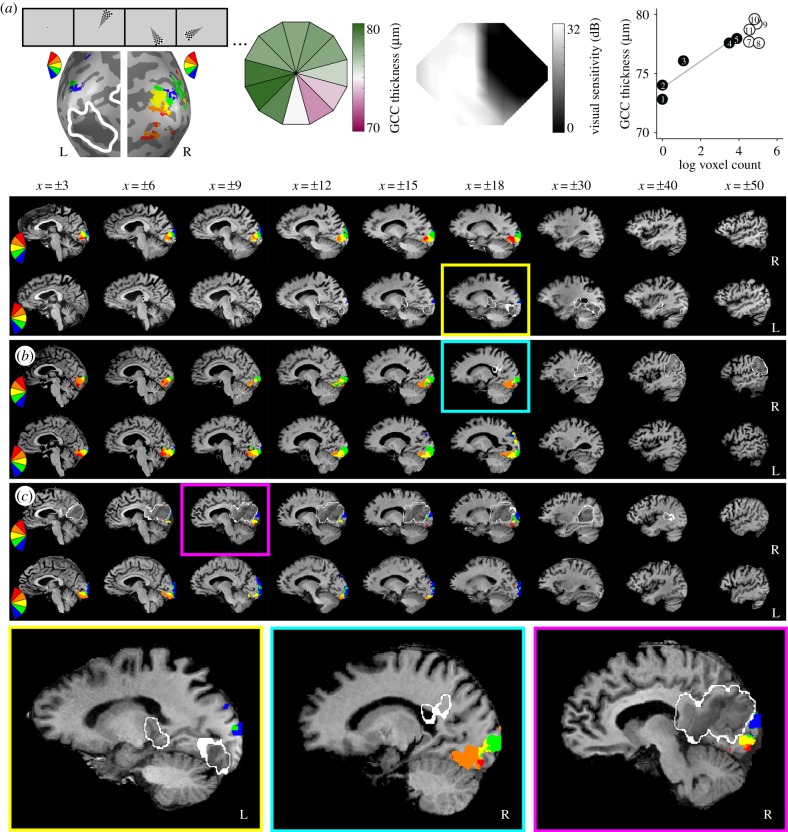
Overview of key measures. (*a*) Example measures from participant 5 collected at the final time point. Winner map of fMRI activity to flickering checkerboard wedges (stimulus example shows random order, lesion outlined from clinical T2 FLAIR or diffusion-weighted image *DWI shown in white; left panel), GCC thickness averaged over both eyes (shown in retinal coordinates; left middle panel), visual field cut (black is blind) determined from automated 24–2 Humphrey perimetry collected as standard of care two days post-stroke (right middle panel) and a plot of the relation between GCC thickness and fMRI activity binned by initial blind (black circles, TD ≤ −6 dB) or sighted wedge locations (white circles, TD > −6 dB; right panel). In the plot, GCC thickness values are translated to visual field coordinates (flipped horizontally); numbers correspond to the wedge number by clock hour, grey line is for blind wedges only. Sagittal slices showing the lesion (outlined in white) and a winner map of visual cortex activity, masked by the medial occipital lobe, for representative patients of varying lesion volumes: (*a*) small (participant 5, final time point), (*b*) medium (participant 15, initial time point), and (*c*) large (participant 14, initial time point). See electronic supplementary material, figure S2 for all other participant activation maps.

The goal of the current investigation was to understand whether trans-synaptic retrograde degeneration of retinal ganglion cells is related to visually evoked activity in the early visual cortex. The key test consists of whether additional variance in GCC thickness can be explained when we use a neural measure of visual ability (fMRI retinotopy) in addition to a behavioural measure (perimetry). The neural measure subsumes the behavioural measure, in that visual ability at sighted locations in the visual field should always be represented by visual cortex activity. In order to compare neural activity with visual sensitivity and GCC thickness, we divided the Humphrey visual field and the macular OCT data into the same 12 polar angle ‘wedges’ of the visual field corresponding to the fMRI stimuli (see Methods, figure 1; electronic supplementary material, figure S2).

Before turning to the critical test of our hypothesis, we first establish two key findings in our dataset replicating prior studies: (i) there is a relation between the spatial extent of GCC thinning and the visual defect, and (ii) there is stimulus-evoked activity in the early visual cortex for stimuli presented in phenomenally blind areas of the visual field.

### (a) Ganglion cell complex thinning matches the field cut and follows the anatomy of the lesion

Our full dataset replicates the prior finding that patients with visual field deficits secondary to stroke have reduced GCC thickness as a function of time since stroke (*r*^2^ = 0.28, *t*_13_ = −2.85, *p* = 0.014). We also replicate the finding that there is a spatial correlation between GCC thickness and total deviation (TD; from Humphrey perimetry) at the last time point tested in each participant in the full dataset (*r*^2^ = 0.1, *t*_136.5_ = 7.49, p≪0.001).

**Figure 2.**
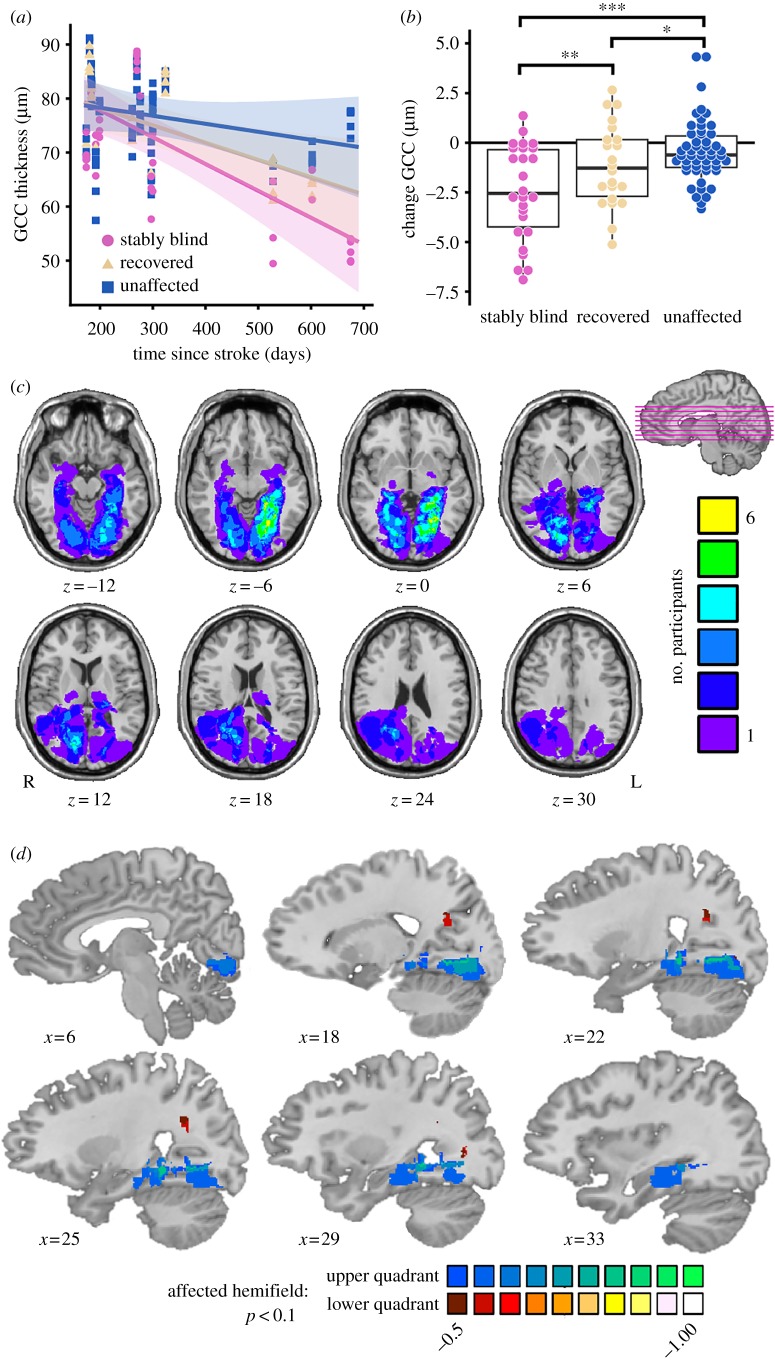
Effect of lesion location, time since stroke, and visual ability on GCC thickness. (*a*) GCC thickness decreases in the stably blind (pink circle) and recovered (yellow triangle) but not unaffected (blue square) areas of the visual field as a function of time since stroke (*n* = 15), error is 95% confidence interval. (*b*) Box plot shows the change in GCC thickness is greatest in stably blind areas of the visual field followed by recovered areas of the visual field (*n* = 10, *p < 0.05, **p < 0.01, ***p < 0.001). (*c*) Lesion overlap map of all participants (*n* = 15) constructed from the acute clinical T2 FLAIR or diffusion-weighted images. (*d*) Voxel-based lesion-symptom map of the change in GCC thickness (*n* = 10) in the upper (blues) or lower (reds) quadrant of the affected hemifield (electronic supplementary material). Lesions in the ventral V1 and/or Meyer's loop were associated with greater GCC thinning in the part of the retina that corresponds to the upper visual field; lesions in Baum's loop were associated with greater GCC thinning in the part of the retina that corresponds to the lower visual field (*p* < 0.1 two-tailed, colour bar is *r* value for the point-biserial correlation between presence/absence of a lesion and change in GCC thickness).

Since all patients received Humphrey perimetry testing as standard of care within the first two months post-stroke, we were able to classify visual field ‘wedges’ as stably blind (TD ≤ −6 dB (blind) at the first and last time points), recovered (TD ≤ −6 dB (blind) at the first time point and >−6 dB (sighted) at the last time point), and unaffected (TD > −6 dB (sighted) at the first and last time points). In the full dataset (figure 2*a*), both stably blind (*t*_14.4_ = −4.31, *p* = 0.001) and recovered wedges (*t*_16.7_ = −2.67, *p* = 0.016) had reduced GCC thickness as a function of time since stroke, whereas unaffected wedges did not (*t*_14.0_ = −1.3, *p* = 0.22). Furthermore, the rate of reduced GCC thickness over time depended on the change in vision (stably blind versus recovered *t*_133.1_ = 3.03, *p* = 0.003; stably blind versus unaffected *t*_131.4_ = 8.15, p≪0.001; recovered versus unaffected *t*_132.7_ = 3.09, *p* = 0.002). Similarly, in the subset of participants for whom we had an initial and a final OCT measure (*n* = 10), thinning in stably blind areas of the visual field was significantly greater than thinning in recovered areas of the visual field (*t*_95.4_ = 2.02, *p* = 0.046) and unaffected areas of the visual field (*t*_90.5_ = 5.64, p≪0.001). Thinning in recovered areas of the visual field was also significantly greater than thinning in unaffected areas of the visual field (*t*_93.7_ = 2.56, *p* = 0.01, figure 2*b*). The novel finding that GCC thinning occurs in the area of the retina corresponding to recovered areas of the visual field suggests that spontaneous visual recovery is not prohibited by, nor does it prevent, retinal ganglion cell degeneration after stroke.

Since the visual field defect is a direct consequence of the location of the lesion, we extended prior observations by using voxel-based lesion-symptom mapping [20] to test whether GCC thickness is spatially related to the ischaemic lesion in a manner consistent with the anatomy of the visual pathway. Figure 2*c* shows the distribution of lesions across all 15 participants. We tested the point-biserial correlation at each voxel between the presence or absence of a lesion (across participants) and the final average GCC thickness in the upper or lower quadrant of the affected hemifield (across participants). The resulting maps indicate where lesion presence is related to GCC thinning. These maps recapitulated the retinotopic organization of the visual system at the level of the optic radiations and early visual cortex (figure 2*d*). While it is the case that, at the extreme, damage to all of early visual cortex would preclude *any* visually driven cortical activity, only two of the 15 patients we studied were missing the entirety of early visual cortex in one hemisphere (participants 3 and 13). The partial preservation of early visual cortex in 13 out of 15 patients opens up the possibility of testing whether early visual cortex activity in response to stimuli presented in the blind field is related to GCC thickness.

### (b) Early visual cortex activity can be evoked by stimuli presented in the blind field

Prior studies have shown that some patients have preserved early visual cortex activity in response to stimuli presented in their blind field [11–18]. That phenomenon is also present in our data: electronic supplementary material, figure S3 shows that over 50% of participants had substantial visual cortex activity in response to at least one wedge presented in a blind area of their visual field (blind voxels) and this observation was present across a range of statistical thresholds used to define significant activity over baseline. The number of active voxels across blind wedge locations and across participants varied widely at a given time point (electronic supplementary material, figure S4), but the ‘blind voxel’ count did not vary over time (see electronic supplementary material). In subsequent analyses, we took the natural log of the voxel count in order to reduce the degree of inter-subject variability in raw voxel counts (electronic supplementary material, figures S4 and S5).

### (c) Preservation of ganglion cell complex thickness is related to visual cortex activity in the blind field

**Figure 3.**
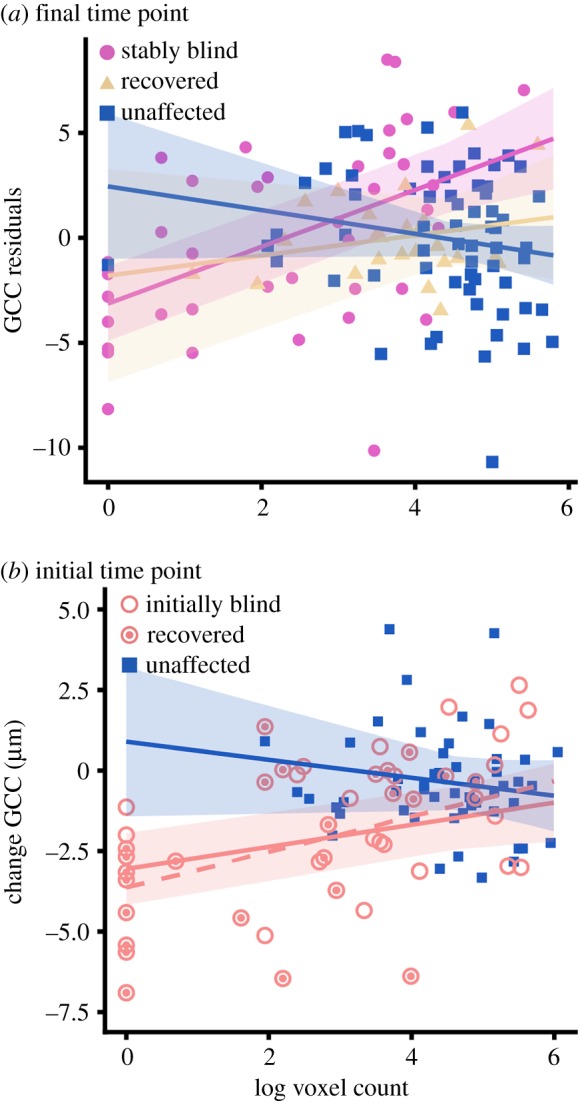
GCC thickness is associated with visual cortex activity. (*a*) GCC thickness at the final time point, controlling for change in visual ability over time, is related to the number of voxels in which there is stimulus-evoked activity for stably blind areas of the visual field at the final time point (*n* = 13; pink circle, stably blind; yellow triangle, recovered; blue square, unaffected). (*b*) The number of voxels that respond to stimulation of the initial blind field (red circle) but not the unaffected areas of the visual field (blue) at the initial time point (less than two months post-stroke) predicts the change in GCC thickness at the final time point (five+ months post-stroke, *n* = 10). This relation remains when wedge locations that recover vision are removed from the analysis (dashed red line; open red circle, recovered; filled red circle, stably blind). Error is 95% confidence interval. (Online version in colour.)

With the replication of both GCC thinning in blind areas of the visual field and preservation of early visual cortex activity in the phenomenal blind field, we next tested the hypothesis that visual cortex activity is related to GCC thickness. In order to test this hypothesis, we modelled final GCC thickness as a function of the number of active voxels per wedge, controlling for change in visual ability over time (*n* = 13). This model accounted for significantly more variance in GCC thickness than the model without fMRI activity (*r*^2^ = 0.43 versus *r*^2^ = 0.40, *χ*^2^ = 23.54, p≪0.001). The relation between final GCC thickness and final fMRI activity in stably blind areas of the visual field was significantly different from that for unaffected areas of the visual field (*t*_111.4_ = −3.85, *p* < 0.001), but not different from that for recovered areas (*t*_111.4_ = −1.35, *p* = 0.18). In stably blind areas of the visual field, the greater the amount of activity in the early visual cortex at the final time point, the less GCC thinning was present at that final time point (*t*_112.4_ = 4.62, p≪0.001). Importantly, that same relation was not present for recovered (*t*_111.4_ = 0.69, *p* = 0.49) or unaffected areas of the visual field (*t*_113.4_ = −0.87, *p* = 0.39; figure 3*a*). For stably blind areas of the visual field, visual cortex activity accounted for 6% of the variance in GCC thickness over and above time since stroke (*r*^2^ = 0.54 versus *r*^2^ = 0.48, *χ*^2^ = 7.76, *p* = 0.005). We found a similar relation (*t*_30.2_ = 2.24, *p* = 0.03) when relating GCC thickness, controlling for time since stroke, with the ratio between the logs of the numbers of active voxels that responded to a wedge in the blind visual field and to the mirror-image wedge in the unaffected hemifield (electronic supplementary material, figure S4D).

Since lesion size was correlated with GCC thickness (see electronic supplementary material for discussion and data in figure S6), we added the interaction between lesion size and change in visual ability to the model. This model accounted for significantly more variance in GCC thickness than the model without lesion size (*r*^2^ = 0.63 versus *r*^2^ = 0.43, *χ*^2^ = 12.88, *p* = 0.005). Importantly, however, the relation between visual cortex activity and GCC thickness was significant for blind areas of the visual field when controlling for the interaction between lesion size and change in visual ability (*t*_112.4_ = 3.98, *p* < 0.001). These findings were also robust to a stricter threshold for defining which wedges of the visual field were blind (electronic supplementary material). Finally, these findings do not depend on the statistical threshold for significant voxel activity (*α* = 0.001) as the relation between visual cortex activity and GCC thickness in blind areas of the visual field was significant across a range of *α* levels from 0.1 to 0.0000005 (electronic supplementary material, figure S7A).

### (d) Initial visual cortex activity is related to subsequent ganglion cell complex thinning

Next, we asked whether activity in the early visual cortex protects against GCC thinning. In order to test this hypothesis, we modelled the *change* in GCC thickness as a function of initial fMRI activity and initial visual ability in the subset of participants who had a study visit within the first two months post-stroke (*n* = 10). Since the slope describing change in GCC thickness over time was modulated by initial visual ability (*t*_88.6_ = 3.52, *p* < 0.001), we included the interaction between time since stroke and initial visual ability in the model relating change in GCC thickness to initial fMRI activity. Initial fMRI activity accounted for significantly more variance in GCC thickness than the model without fMRI activity (*r*^2^ = 0.28 versus *r*^2^ = 0.22, *χ*^2^ = 8.15, *p* = 0.02). The relation between initial fMRI activity and change in GCC thickness for the initial blind field was significantly different from that for unaffected areas of the visual field (*t*_92.3_ = −2.24, *p* = 0.03). In the initial blind field, greater visual cortex activity at the first time point was associated with less GCC thinning at the final time point (*t*_93.2_ = 2.54, *p* = 0.01). Importantly, the relation between initial visual cortex activity and change in GCC thickness was not significant in the unaffected regions of the visual field (*t*_92.1_ = −1.15, *p* = 0.25, figure 3*b*). For the initial blind field, initial visual cortex activity accounted for 10% of the variance in change in GCC thickness, over and above time since stroke (*r*^2^ = 0.3 versus *r*^2^ = 0.2, *χ*^2^ = 5.78, *p* = 0.02). This relation was even more robust when recovered wedges were removed from the dataset (*t*_67.2_ = 3.13, *p* = 0.003), suggesting that the relation between GCC thickness and visual cortex activity in the initial blind field is not driven by visual recovery. In addition, the relation was not significant when change in GCC thickness was replaced with initial GCC thickness (*t*_38.8_ = 0.18, *p* = 0.86), which confirms the importance of the temporal relation between visual cortex activity and GCC thinning.

As follow-up, we found that the relation between initial visual cortex activity and subsequent GCC thinning was significant for the original blind field when controlling for the interaction between lesion size and initial visual ability (*t*_91.7_ = 2.53, *p* = 0.01). These findings were also robust to a stricter threshold for defining which wedges of the visual field were blind (electronic supplementary material). Finally, the significance of these findings did not depend on the *α* threshold for defining significant visual cortex activity (*α* = 0.001) as we continued to observe a significant relation between initial visual cortex activity and change in GCC thickness at a range of thresholds from *p* < 0.1 to *p* < 0.0005 (electronic supplementary material, figure S7B).

## 3. Discussion

Our study of patients with visual field defects secondary to stroke examined retinal ganglion cell degeneration in the context of preserved early visual cortex activity for stimuli presented in the blind field. We found that GCC thickness in stably blind areas of the visual field is related to the amount of visual cortex activity representing that area of the visual field. Furthermore, the amount of visual cortex activity that is present in the first weeks to months post-stroke predicts GCC thinning measured five+ months after stroke. While there are many possible reasons for there to be a dissociation between the absence of phenomenal vision and the presence of visual cortex activity (see electronic supplementary material for further discussion), it is important to emphasize that our core conclusion—GCC thinning is activity dependent—follows independently of the underlying cause of the early visual cortex activity despite phenomenal blindness.

### (a) Retinal ganglion cell health relies on early visual cortex activity: inferring causation from what are inherently correlational data

Our main finding stems from a prospective observational study that relies on correlations among independent measures (e.g. OCT, fMRI, measures of visual ability). One possible objection is that direct causal evidence has not been presented. A valuable framework within which to carefully consider the evidentiary status of causal claims based on the data we have reported is provided by Hill's criteria for causation [21].

— Criterion 1—*what is the strength of the effect?* Our full model, which includes time since stroke, change in visual ability, and visual cortex activity, accounts for 43% of the variance in final GCC thickness when using final visual cortex activity (*r* = 0.66, large effect size) and 28% of the variance in the change in GCC thickness when using visual cortex activity soon after the stroke (*r* = 0.52, large effect size).
— Criterion 2—*what is the specificity of the effect?* The burden of our argument was to show that there is variance unique to visual cortex activity in the blind field that is not attributable to other sources: 6% of the variance in GCC thickness and 10% of the variance in change in GCC thickness was *uniquely* explained by visual cortex activity in the stably blind/initial blind field, respectively.
— Criterion 3—*does the putative cause precede the effect?* In our longitudinal analyses, initial visual cortex activity preceded (by approx. four months) the subsequently measured change in GCC thickness. Importantly, the relation between initial visual cortex activity and GCC thickness was not present for GCC measures taken at the same time as the measure of initial visual cortex activity.
— Criterion 4—*does greater exposure generate a greater effect?* The relation between GCC thickness and visual cortex activity was explained by a log-linear function.
— Criterion 5—*is there a biologically plausible mechanism?* The survival of retinal ganglion cells could be explained by the retrograde [22,23], activity-dependent release [24,25] of brain-derived neurotrophic factor (BDNF), which has been shown to be an important mediator of retinal ganglion cell survival [26], especially when applied to V1 in addition to the retina [27,28]. Furthermore, BDNF from the superior colliculus is believed to not be sufficient to protect ganglion cells [23]. Thus, the loss of BDNF release due to decreased visual cortex activity could lead to retinal ganglion cell atrophy.

Fulfilment of this subset of Hill's criteria strengthens the inference that the integrity of retinal ganglion cells depends, causally, on the early visual cortex activity.

### (b) The fate of retinal ganglion cells for recovered areas of the visual field

Interestingly, we found that GCC thinning occurred in recovered areas of the visual field, although to a lesser extent than in stably blind areas. We did not, however, detect a relation between visual cortex activity and GCC thickness in recovered areas of the visual field. It may be that because recovered areas of the visual field are often in close proximity to stably blind areas, the part of the retina that corresponds to recovered areas of the visual field also suffers from a decrease in trophic factors. Alternatively, retinal ganglion cell atrophy in recovered areas of the visual field could be a sign of plasticity, because rewiring in the geniculostriate pathway would restore the feed-forward flow of visual information but may not affect the pathways involved in the retrograde transport of trophic factors. For example, visual recovery secondary to plasticity in extra-geniculostriate pathways such as the middle temporal visual area (area MT) [29,30] or the superior colliculus [31] would not improve the delivery of BDNF from V1 to the retina. Regardless of the explanation, we are still left with the fact that areas of the retina corresponding to recovered areas of the visual field also degenerate, albeit to a lesser extent than areas of the visual field that are stably blind.

## 4. Conclusion

Previously, retinal ganglion cell degeneration was considered through the lens of the lesion and the permanent visual defect. We show that visual cortex activity explains unique variance in GCC thickness across the macula of patients with cortical blindness. Since all sighted areas of the visual field have visual cortex activity, we suggest that the superordinate variable is activity in the early visual cortex, rather than phenomenal visual ability. This conceptually reframes the prior literature by emphasizing a process of activity-dependent trans-synaptic retrograde degeneration of retinal ganglion cells, and motivates consideration of whether a similar process may occur in the setting of other pathways. Future visual rehabilitation research should investigate what activity-dependent factors protect against retinal ganglion cell degeneration secondary to geniculostriate pathway damage. In addition, further research should determine whether visual retraining [32] may be more successful when targeting areas of the blind field where GCC thinning has not occurred, because these are more likely to be areas with preserved visual cortex activity. We suggest that measures of visual cortex activity may provide a key piece of information for understanding heterogeneity in GCC thinning, visual recovery, and ultimately, responsivity to visual retraining.

## 5. Methods

### (a) Participants

Stroke patients were recruited through Strong Memorial Hospital (University of Rochester Medical Center) and Rochester General Hospital (Rochester Regional Health) as part of a stroke recovery study approved by the University of Rochester (RSRB #28991, #58133). Inclusion criteria consisted of an MRI-confirmed ischaemic stroke resulting in an isolated homonymous visual field deficit. Exclusion criteria consisted of a National Institutes of Health Stroke Scale score greater than five, a premorbid modified Rankin Scale score greater than two, premorbid monocular or binocular visual field deficits, premorbid retinopathy or optic neuropathy, or history of angle-closure glaucoma or elevated intraocular pressure.

### (b) Procedure

All participants completed automated 24–2 Humphrey perimetry testing for each eye (Zeiss HFAIIi, SITA standard perimetry using a size III white target, fixation enforced, corrected for near vision), as standard of care an average of 13 days post-stroke (range = 2–63 days). At the first study visit, participants received a full neuro-ophthalmologic exam. All study visits included Humphrey perimetry for each eye, spectral-domain macular OCT for each eye (Zeiss Cirrus HD-OCT model 5000, 512 × 128 scan protocol), and binocular fMRI retinotopic mapping (with the exception of participant 4 who did not complete fMRI at any time point, electronic supplementary material, table S1).

### (c) Functional magnetic resonance imaging

The primary location for scanning prior to hospital discharge was at Strong Memorial Hospital (3 T GE 750 W scanner, 8-channel head coil). The primary location for outpatient scanning was the Rochester Center for Brain Imaging (3 T Siemens Trio scanner, 12-channel head coil or a 3 T Siemens Prisma scanner, 20-channel head coil). MRI acquisition protocols were developed in consultation with a neuroradiologist to optimize equivalence in parameters across scanners. In addition, we scanned four healthy control participants across multiple scanners in order to be able to formally compare temporal signal-to-noise and sensitivity to detect retinotopic preferences across scanners. The results of those analyses are reported in detail in electronic supplementary material, table S2 and figure S8; all scanners had more than sufficient signal-to-noise and sensitivity to detect retinotopic preferences.

Visual stimuli during fMRI were presented through binocular goggles regardless of scanner location (Nordic Neuro Lab, Bergen, Norway). The visual field subtended by the goggles was 30° horizontal × 22.5° vertical visual angle. Participants fixated on a central point while a series of black and white checkerboard wedges flickered on a mean luminance background at 5 Hz. Each wedge subtended 11.25° of visual angle, had a central angle of 30° (figure 1*a*) and was presented at each of 12 non-overlapping locations five times per run. Except for a few instances of technical difficulties, fixation was monitored with an eye tracker during all fMRI runs (Arrington ViewPoint; see electronic supplementary material, table S1). Participants completed two runs of the polar angle mapping experiment at each time point and two runs in which they maintained central fixation while a flickering full-field checkerboard disc (diameter = 11.25°) was presented on a mean luminance background for 2 repetition time (TRs), five times per run; stimuli were separated by 14 TRs of a full-field mean luminance background. Voxels that significantly responded to a given contralateral wedge location (wedge of interest > mean luminance baseline, *p* < 0.001) were identified within an independently defined mask of early visual cortex (electronic supplementary material). For visualization purposes, interpolated winner maps were constructed from the contralateral wedge location with the largest *t*-value at each voxel, thresholded at *p* < 0.001 (figure 1; electronic supplementary material, figure S2).

### (d) Aligning optical coherence tomography, Humphrey perimetry, and functional magnetic resonance imaging data

OCT measurements for both eyes were extracted from data points within an elliptical annulus mask centred on the fovea (inner radii: 0.5 mm vertical × 0.6 mm horizontal, outer radii: 2 mm vertical × 2.4 mm horizontal). The data were then automatically segmented [33,34] to isolate the GCC. Segmentation quality was checked by C.L.S. and instances of poor segmentation were further inspected by Z.R.W. (who was kept blinded to other results at the time of inspection). GCC thickness data points were averaged across both eyes and flipped across the horizontal meridian in order to align visual field and retinal coordinates. Humphrey perimetry data (TD) for both eyes were combined into a winner map using an in-house script (MATLAB), and following the precedent set in [35]. GCC thickness and TD measures were averaged for all test points within each ‘wedge’ (based on a division of the visual field into the 12 non-overlapping fMRI polar angle stimuli). The wedges at 12 o'clock and 6 o'clock were excluded from all analyses due to nasotemporal overlap of the ganglion cell projections [36].

Wedges were defined as sighted (average TD > −6 dB) or blind (average TD ≤ −6 dB) at each time point. This classification was based on the elbow of the cumulative distribution plot of all TD values for all participants at all test locations and time points (electronic supplementary material, figure S9). Furthermore, because all of our participants received Humphrey perimetry as standard of care after their stroke, we were able to classify the change in visual ability of each wedge as stably blind (TD ≤ −6 dB (blind) at the first and last time points), recovered (TD ≤ −6 dB (blind) at the first time point and > −6 dB (sighted) at the last time point) and unaffected (TD > −6 dB (sighted) at the first and last time points).

### (e) Statistical analyses

Statistical analyses were conducted with R Studio (lme4, lmerTest, effects, and MuMIn). All statistical tests used an *α* = 0.05 to determine significance and were two-tailed, stepwise mixed-effects linear regressions with participant as the random effect on the intercept. Degrees of freedom were based on the number of wedges and were estimated with the Satterthwaite approximation. The reported *R*^2^ values are the marginal *R*^2^ values, which represent the variance explained by the fixed effects.

## Supplementary Material

Electronic Supplementary Material

## Data accessibility

Summaries of all processed data included in the statistical analyses plus raw MRI (BIDS format [37]), OCT, Humphrey Visual Field, and Letter Test data are available online (Carnegie Mellon University repository KiltHub through: https://doi.org/10.1184/R1/7403780.
