# Supplementary material for: Survival of retinal ganglion cells after damage to the occipital lobe in humans is activity dependent
Source: Proc Biol Sci. 2019 Feb 27;286(1897):20182733. doi: 10.1098/rspb.2018.2733 (PMC6408898; doi:10.1098/rspb.2018.2733)
Supplement: Electronic Supplementary Material [file rspb20182733supp1.pdf]

Electronic Supplementary Material for

Survival of Retinal Ganglion Cells After Damage of the Occipital Lobe in Humans is Activity Dependent

Colleen L. Schneider[a,b,c], Emily K. Prentiss[d], Ania Busza[d], Kelly Matmati[e], Nabil Matmati[e], Zoë R. Williams[d,f,g], Bogachan Sahin[d], Bradford Z. Mahon[c,d,g,h,*]

a) Department of Brain & Cognitive Sciences, University of Rochester, Rochester, NY, USA 14627
b) Medical Scientist Training Program, University of Rochester School of Medicine & Dentistry, Rochester, NY, USA 14642
c) Department of Psychology, Carnegie Mellon University, Pittsburgh PA 15206
d) Department of Neurology, University of Rochester Medical Center, Rochester, NY, USA 14642
e) Department of Neurology, Rochester Regional Health, Rochester, NY, USA 14621
f) Department of Ophthalmology, University of Rochester Medical Center, Rochester, NY, USA 14642
g) Department of Neurosurgery, University of Rochester Medical Center, Rochester, NY, USA 14642
h) Center for Visual Science, University of Rochester, Rochester, NY, USA 14642

Correspondence:
Bradford Z. Mahon, PhD
Email: bmahon@andrew.cmu.edu

This PDF file includes:

Supplementary text
Figs. S1 to S10
Tables S1 to S3
References for SI

Materials and Methods

Participants. We consented 35 stroke patients with frank or de-afferenting visual cortex lesions secondary to ischemic stroke in the territories of the posterior or middle cerebral arteries between April 2015 and July 2017. In order for a patient to be included in the analyses described in this article, s/he had to have at least one study visit with OCT that occurred ≥ 5 months post-stroke (Fig. S1). Fifteen participants completed one study visit at greater than 5 months post-stroke (4 females, mean age = 63.67 years, mean time since stroke = 310 days, range time since stroke = 174-675 days) and 10 of these participants also completed a study visit less than 2 months post-stroke (3 females, mean age = 64.9 years, first time point range = 1-54 days post-stroke, first time point mean = 10.8 days). Of note, participants 11-15 were also enrolled in an ongoing randomized double-blind pilot clinical trial (FLUORESCE, NCT02737930) to determine the efficacy of fluoxetine on visual recovery. At the time of this writing the authors were blinded to the group assignment.

Measuring retinal ganglion cell complex thickness. Retinal health is routinely assessed in the clinic using a non-invasive test called Spectral-Domain Optical Coherence Tomography (OCT) that generates a three-dimensional image of the retina. In order to assess the integrity of retinal ganglion cells, the OCT scan is either centered on the optic disc to measure the thickness of the retinal nerve fiber layer, which is comprised of the ganglion cell axons [1–6], or the fovea to measure the thickness of the ganglion cell complex (GCC) [5–8], which is comprised of the retinal ganglion cell and inner plexiform layers. Here, we focus on macular OCT as an index of ganglion cell health for two reasons: 1) GCC thinning is detected before retinal nerve fiber layer

thinning [7,8]; and 2) we wanted to capitalize on the topographic correspondence between the retinotopic organization of the macula and visual cortex [8].

With the exception of the right eye of four participants (6, 7, 8, and 15), thickness measurements from both eyes were averaged. The right eye of participant 6 was excluded due to poor scan quality at the final visit (quality was 5/10). The right eye of participant 7 was excluded due to nasal thickening. The right eye of participant 8 was excluded due to macular degeneration that was diagnosed after enrollment in the current study. The right eye of participant 15 was excluded due to chronic cystic macular edema. For those participants with monocular pathology or poor scan quality, only the GCC thickness from one eye at each time point was included in the analyses.

We tested the linear relation between GCC thickness and time since stroke because prior models of retinal ganglion cell degeneration suggest that the time range in our study is early enough to be approximated by a linear function; beyond 2 years after stroke, the relation is a negatively accelerating exponential decay function [2,3,9].

MRI acquisition parameters and additional scans. Scanning parameters at locations other than the primary outpatient site (Rochester Center for Brain Imaging) were chosen in consultation with an MR physicist and neuroradiologist in order to be equivalent to the primary scanning location. We further ensured that all scanners produced data with sufficient signal to noise by scanning healthy controls (n = 4) on multiple scanners using the same polar angle mapping protocol as was used in patients. Formal assessments of signal-to-noise across scanners

consisted of calculating temporal signal-to-noise (averaged across both runs of polar angle) and an analysis of the reproducibility of polar angle maps (Table S2, Fig. S7).

Scanning Parameters for 3T Siemens Prisma: MPRAGE (magnetization-prepared rapid acquisition gradient echo) pulse sequence (repetition time (TR) = 2530 ms, echo time (TE) = 3.44 ms, flip angle = 7°, field of view (FOV) = 256 mm, matrix = 256 x 256 x 256, 1 x 1 x 1 mm); BOLD fMRI echo planar imaging pulse sequence (TR = 2200ms, TE = 30ms, flip angle = 90°, FOV = 256 mm, matrix 64 x 64, 33 axial ascending slices interleaved odd-even, isovoxel size 4 x 4 x 4 mm).

Scanning Parameters for 3T Siemens Trim Trio: MPRAGE (magnetization-prepared rapid acquisition gradient echo) pulse sequence (repetition time (TR) = 2530 ms, echo time (TE) = 3.44 ms, flip angle = 7°, field of view (FOV) = 256 mm, matrix = 256 x 256 x 256, 1 x 1 x 1 mm); BOLD fMRI echo planar imaging pulse sequence (TR = 2200 ms, TE = 30 ms, flip angle = 90°, FOV = 256 mm, matrix 64 x 64, 33 axial ascending slices interleaved odd-even, isovoxel size 4 x 4 x 4 mm).

Scanning Parameters for 3T GE 750W: FSPGR BRAVO (fast spoiled gradient recalled acquisition in the steady state brain volume imaging) pulse sequence (TR = 8.5 ms, TE = 3.2 ms, flip angle = 12°, FOV = 256 mm, matrix = 256 x 256 x 256, 1 x 1 x 1 mm; BOLD fMRI gradient echo pulse sequence (TR = 2200 ms, TE = 30 ms, flip angle = 90°, FOV = 256 mm, matrix 64 x 64, 33 axial ascending slices interleaved odd-even, isovoxel size 4 x 4 x 4 mm).

Participants completed one of three variants of the polar angle mapping experiment (Table S1): 1) Continuous: a slowly clockwise rotating wedge; 2) Sequential: a wedge that appeared at each location in a contiguous clockwise order every 2 TRs; or 3) Random: a wedge that randomly appeared every 2 TRs in one of 12 non-overlapping locations with the constraint that no two consecutive wedges were presented sequentially [10]. For each variant, each wedge location appeared 5 times per run.

In addition to two runs of the wedge stimulus and two runs of the full-field flickering checkerboard stimulus, two other scans were acquired at each time point but not analyzed herein: 1) two runs with flickering checkerboard annuli to study eccentricity preferences in visual cortex, and 2) one run of resting state fMRI. At the final scan session, additional scans were collected on a subset of patients on an ad hoc basis as part of a different study.

Fixation was confirmed with an infrared eye tracker (Arrington ViewPoint, see Table S1 for details about eye tracker use). Out of 25 fMRI sessions, only two fMRI sessions were excluded completely from subsequent analyses: the first time point fMRI from participant 5 was excluded due to technical difficulties and the last time point fMRI from participant 15 was excluded because the participant was asleep during both runs. In three other cases, one of the two polar angle runs was excluded because the participant fell asleep (Table S1).

Functional MRI data were analyzed with the *Brain Voyager* software package (Version 2.8), in-house scripts drawing on the BVQX toolbox for MATLAB, and FreeSurfer (http://surfer.nmr.mgh.harvard.edu/) following the same procedures as prior papers from our group [11–13]. Preprocessing of the functional data included slice scan time correction (sinc interpolation), and motion correction with respect to the first volume of the first functional run. Functional data were co-registered to the first time point T1-weighted de-skulled image on an

individual basis in native space. BOLD and anatomical volumes were transformed into Talairach space [14]. No spatial smoothing was applied. Time course data from a given session were combined in a multi-run general linear model. The model consisted of 12 predictors for the 12 wedge locations convolved with a standard 2-gamma hemodynamic response function and 6 predictors of no interest to attract variance from volume-to-volume change in head position. Eye tracking data was used to exclude runs where the participant fell asleep or had poor fixation (Table S1).

Definition of visually active voxels. Participant-specific masks for early visual cortex were created by taking the intersection of the participant's anatomical mask of medial occipital lobe and the participant's functional map for visually responsive voxels using the full-field checkerboard stimulus. Specifically, an anatomical mask of medial occipital lobe was defined within one functional voxel from the right- or left-most border of the calcarine sulcus, as viewed in the coronal plane (Table S2). In the case of complete destruction of the calcarine sulcus by the stroke lesion, the intact hemisphere coordinates were flipped across the mid-sagittal line. In addition, a functional mask of visually-responsive cortex was independently defined based on the anterior-most border of visually-responsive voxels using the full-field checkerboard stimulus data from the last time point in each participant (contrast of checkerboard on > mean luminance baseline, $p < 0.05$). We flipped the functional mask in each hemisphere across the mid-sagittal line and took the union of the two masks to generate two symmetrical visually-responsive cortical masks, separated by hemisphere, for each participant. We then took the intersection of the anatomical medial occipital lobe mask and the functional visually-responsive cortex mask to generate a medial occipital lobe visual cortex mask that was symmetric about the mid-sagittal

line; this mask was down sampled to 3mm^3 voxels to align with functional space. The number of significantly active voxels for each wedge location within the independently masked visually-responsive medial occipital lobe was used as a measure of neural representation of vision at each time point [15]. With these voxel counts, we tested the logarithmic relation between GCC thickness and the natural log of the number of active voxels based on initial visualization of the data (Fig. S9, wedges with a count of 0 were changed to 1 in order to take the logarithm).

Lesion mapping. The clinical T2 FLAIR or diffusion weighted scan collected in the acute stroke phase was used to draw a lesion mask for each patient, using Clusterize, a semi-automatic lesion segmentation toolbox for SPM [16]. The clinical T2 FLAIR or diffusion weighted scan was co-registered to the T1 anatomy collected at the first time point in each patient and the transformation matrix was applied to the lesion mask. In order to create the voxel-based lesion symptom maps in Fig. 2D, the change in GCC thickness for the upper and lower quadrant of the affected hemifield was calculated for each participant with an initial and a final OCT. Point biserial correlations were used to relate change in GCC thickness to the presence/absence of a lesion across the group of participants, at each voxel in the brain [17]. In order to increase power for the voxel-based lesion-symptom mapping analysis, all lesion masks were projected into the right hemisphere and all visually-affected hemifields were correspondingly projected into the left visual hemifield.

Letter detection and identification task. To test high contrast vision within the central 22.5 degrees, we used a letter identification visual field test that has been previously published in our lab [12,18]. Briefly, black letters were presented one at a time on a mean luminance background

for 133 ms in one of 72 randomly-ordered locations (Fig. S10). With both eyes open, participants identified each letter out-loud as it was presented. Responses were recorded with a microphone. Correct identification was awarded 1 point, detection with incorrect identification was awarded 0.5 points, and no detection (missed) was not awarded any points. Performance for the area of the visual field subtended by each wedge was calculated by averaging the performance for all letters presented in the area covered by that wedge, collapsed across eccentricity. There was no formal eye tracking when participants were tested in the hospital with the letter test; however, one experimenter watched the participant's eyes during the testing and noted any breaks from fixation. All outpatient visits were conducted in our lab with formal eye tracking (table mounted EyeLink 1000, desktop mode).

Results

Size of lesion within early visual cortex is related to GCC thinning in stably blind areas of the visual field. It is likely that there is a relation between GCC thinning and lesion size, as patients sustaining larger lesions may tend to have more early visual cortex or subcortical damage, and thus a greater extent of retinal ganglion cell degeneration in the blind field. In line with that expectation, we found that total lesion size was correlated with GCC thickness in stably blind areas of the visual field ($t(144) = -1.99$, $p = 0.048$, Fig. S5A). We separately tested how lesion size in early visual cortex versus outside of early visual cortex is related to GCC thickness. One possibility is that larger extrastriate lesions (lesion size outside the early visual cortex mask) would be associated with greater GCC thinning, as such lesions may tend to affect the optic radiations, which are closer along the visual pathway to the retina than primary visual cortex. Another possibility is that lesion size in early visual cortex is most directly related to GCC

thinning. The results for areas of the retina that corresponded to stably blind areas of the visual field indicated no relation between GCC thickness and extra-striate lesion size ($t(134.0) = -0.28$, $p = 0.78$, Fig. S5B), and a significant correlation between early visual cortex lesion size and GCC thickness ($t(134.0) = -4.42$, $p << 0.001$, Fig. S5C). We note that the lack of a relation between extra-striate lesion size and GCC thinning should be revisited with future studies explicitly designed to test this important issue. At a minimum though, these findings suggest lesion size in early visual cortex is directly related to GCC thinning.

Number of blind voxels does not change over time. When we considered the number of significantly active voxels for all participants and fMRI sessions, we found that the number of voxels did not change over time as a function of change in vision (stably blind versus recovered: $t(443.2) = 0.95$, $p = 0.34$; stably blind versus unaffected: $t(435.9) = 1.40$, $p = 0.16$; recovered versus unaffected: $t(439.6) = -0.03$, $p =0.97$) nor was there a main effect of time on the number of significantly active voxels ($t(108.8) = -0.77$, $p = 0.44$). Collapsing across all participants and time points, there was a significant difference in the number of active voxels representing stably blind versus unaffected wedges (mean stably blind = 55.2 voxels, mean unaffected = 110.2 voxels, $t(439.0) = 7.23$, $p << 0.001$) and recovered versus unaffected wedges (mean recovered = 74.1 voxels, $t(446.0) = 3.51$, $p < 0.001$), but only a trend comparing stably blind and recovered wedges ($t(445.7) = -1.73$, $p = 0.08$).

Relation between GCC thickness and visual cortex activity is robust to definition of blindness. Since the raw total deviation values in what was defined as the blind field ranged from -32.67 dB to -6.33 dB, it is possible that blind wedges with high early visual cortex activity

are also those with less negative total deviations. If this were the case, then the significant relation that we observed between visual cortex activity and GCC thickness in the stably blind field may be due to the variability in total deviation in the blind field. Indeed, the correlation between final total deviation and visual cortex activity was significant for the stably blind field ($t(123.0) = 3.66$, $p < 0.001$) but not recovered ($t(123.0) = -0.49$, $p = 0.63$) or unaffected areas of the visual field ($t(124.0) = -0.44$, $p = 0.66$). When we added final total deviation to the model, however, we found that the relation between early visual cortex activity and GCC thickness remained significant in the stably blind field ($t(111.2) = 3.75$, $p < 0.001$), suggesting that greater visual cortex activity is associated with greater GCC thickness even for densely blind areas of the visual field.

We further tested whether our main finding was robust to the criterion used for defining blind wedges. We re-analyzed our data by classifying wedges as blind if the average sensitivity in that wedge was less than 10 dB. This definition of blindness was based on "The Guide for the Evaluation of Visual Impairment", which defined blind visual field test locations as those with a sensitivity less than 10 dB [19] and a natural history study of visual recovery in stroke patients with homonymous visual field defects, which used Goldmann perimetry with varying sizes of 4e isopters (equivalent to a sensitivity of 10 dB on Humphrey perimetry) [20]. Using this alternative definition of blindness (sensitivity < 10dB) did not alter the main finding that there is a significant correlation at the final time point between visual cortex activity and retinal ganglion cell thickness that is specific to stably blind areas of the visual field (stably blind: $t(124) = 2.85$, $p = 0.005$; recovered: $t(124) = 0.21$, $p = 0.84$; unaffected: $t(124) = -1.02$, $p = 0.31$). This alternative definition of blindness also did not alter the finding that initial visual cortex activity in response to stimulation of the original blind field could predict later GCC thinning (originally

blind: $t(90.1) = 2.23$, $p = 0.03$; unaffected: $t(92.9) = 1.39$, $p = 0.17$).

Discussion

Blind voxels. To date, many fMRI studies of stroke patients have shown early visual cortex activity for stimuli presented in the blind field. In our study, more than half of the participants had at least 50 significantly active voxels for a wedge located in their initial blind field. We refer to such voxels as 'blind voxels.' Blind voxels maintained a clear retinotopic organization. Eye movements cannot explain this phenomenon, because fixation was enforced using an eye tracker (Table S1). Below we enumerate possible explanations for the existence of 'blind voxels'.

- Spared islands of vision [21–24]. One concern that may be raised is that the clinical measure of vision (24-2 Humphrey perimetry) is a relatively coarse measure of visual ability, and there could thus be 'islands' of spared vision interspersed within the area of the visual defect. This is an important alternative to consider, as the area stimulated during the fMRI experiment was smaller than the area tested with Humphrey perimetry (radius of field of visual stimulation during fMRI = 11.25°). We prospectively addressed this in the design of the study by having all participants also complete a letter detection and identification task with test locations that covered the same retinotopic coordinates as the fMRI wedge stimuli [12,18] (Fig. S10). GCC thickness was still significantly related to visual cortex activity for stimulation of the blind visual field when using performance on the letter detection and identification task as the measure of visual ability ($t(111.7) = 3.31$, $p = 0.001$). This finding indicates that the activity-dependence of GCC thinning cannot be explained by residual vision.

- Neural feedback propagating from higher order visual areas [25], through inter-hemispheric transfer [26,27], or through mental imagery and visual illusions that drive activity in early visual cortex [28,29]. An important concern that may be raised is that participants are able to anticipate the next location of the retinotopic mapping stimulus, and thus drive activity in early visual areas based on expectations of where the stimulus will be (even if those visual areas do not receive direct inputs from the retina). However, we can decisively put this concern to rest because we used a random presentation scheme for the fMRI wedge stimuli specifically to reduce such anticipatory strategies and consequent feedback from higher-order visual areas to early visual cortex.
- As a final set of possibilities, we suggest that a combination of two factors may contribute to the phenomena of 'blind voxels'. First, it could be that the information content of immediately peri-lesional areas is degraded such that while one can still detect visual cortex activity and this activity is sufficient to drive trophic support of retinal ganglion cells, the information driving the activity is too impoverished to support perception. Second, some lesions will disconnect early visual cortex from downstream regions (V2, V3, V4, etc.) – thus preventing visual information in primary visual cortex from being processed further.

Figure S1: Participant recruitment.

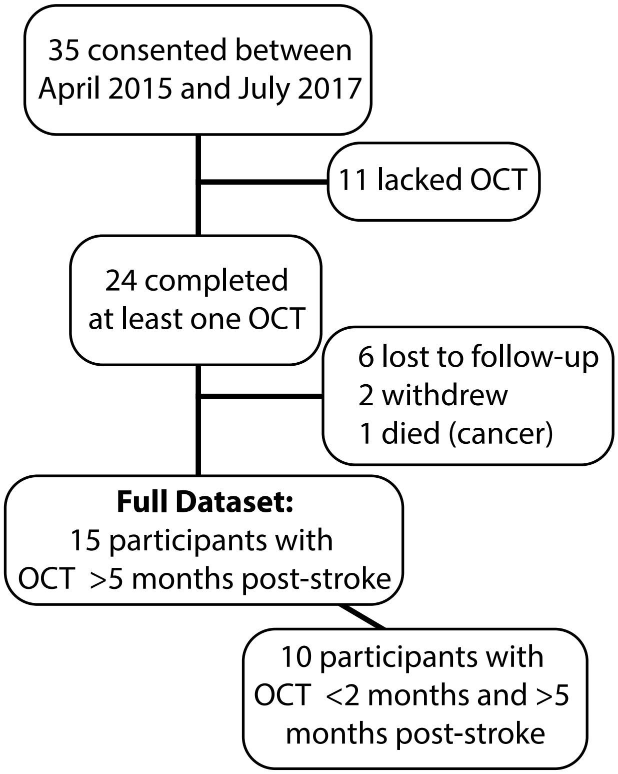

Figure S2: fMRI results. Interpolated winner maps of the retinotopic organization of visual cortex, pseudocolored by the contralateral wedge that elicited the strongest response for a given voxel, thresholded at $p < 0.001$, and masked by visually-responsive medial occipital lobe. Lesions, as determined by the participant's acute clinical T2 FLAIR or DWI, are shown outlined in white. Neural activity is overlaid on sagittal slices of each participant's T1 anatomy. The sole time point is shown for participants 1 – 6 and initial time point is shown for participants 7 – 13. In some cases, activity appears inside the lesion boundary (for example participant 2). This discrepancy occurs because the clinical imaging used to construct the lesion boundary also reflects edema that may or may not evolve into frank tissue damage. Careful inspection of the underlying T1 anatomy (from a chronic time point) reveals that all regions of significant fMRI activity overlie intact tissue. See Fig. 1 for participants 5, 14, and 15; participant 4 lacked any fMRI data.

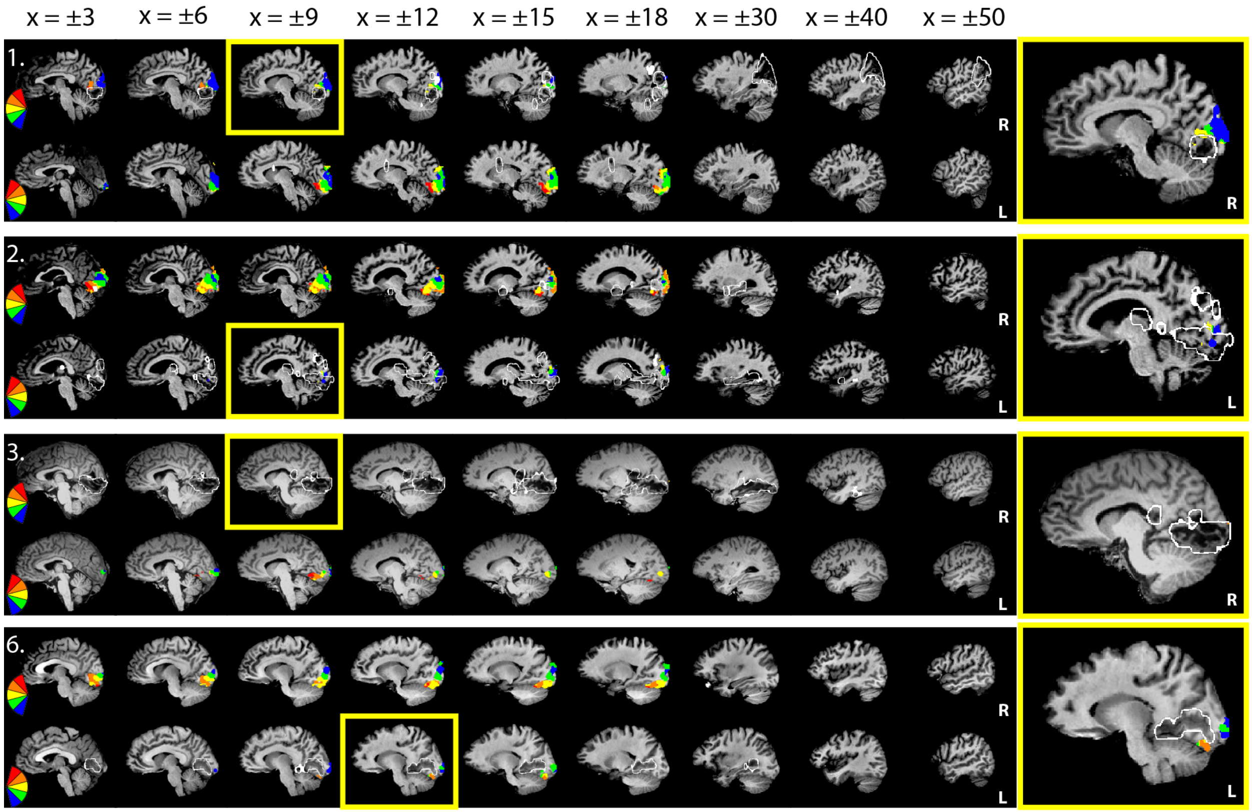

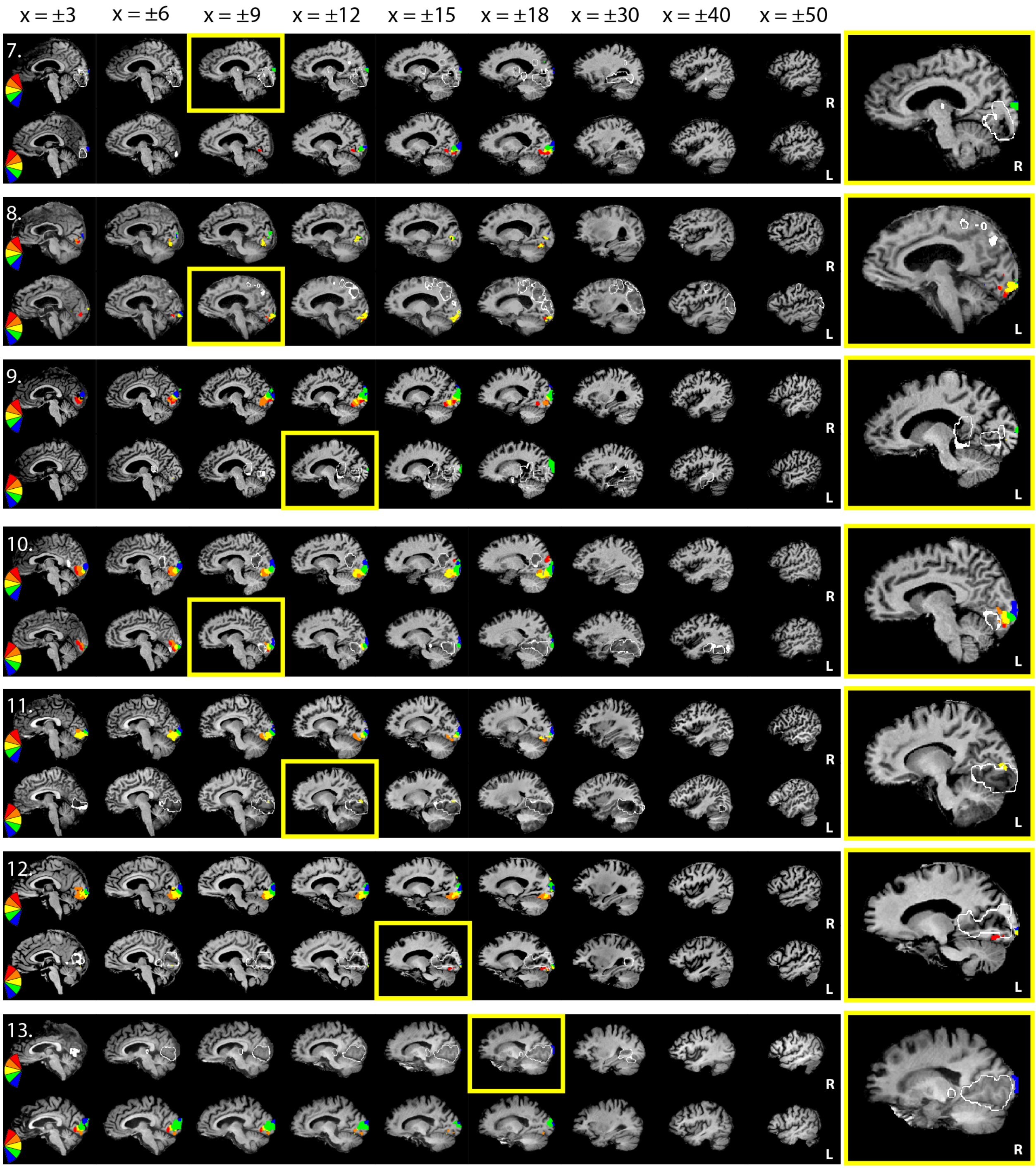
x = ±3
x = ±6
x = ±9
x = ±12
x = ±15
x = ±18
x = ±30
x = ±40
x = ±50
7.
8.
9.
10.
11.
12.
13.
R
L

Figure S3: 'Blind voxel' phenomenon is present for a range of statistical thresholds for defining significantly active voxels. Percent of participants with at least one wedge containing a certain number of significantly active voxels at varying alphas (1: $p < 0.00001$; 2: $p < 0.001$; 3: $p < 0.05$). Lower panel shows area under the curves in the top panel as a function of change in vision and statistical threshold. For ease of viewing, alphas are plotted on a log scale (i.e. alpha = 0.001 is equal to 1×10^{-3} and is therefore plotted on the x-axis at -3). A) Final fMRI binned by change in vision (n = 13), and B) initial fMRI binned by initial vision (n = 10). Pink circle – stably blind; yellow triangle – recovered; blue square – unaffected; red circle – blind at first time point.

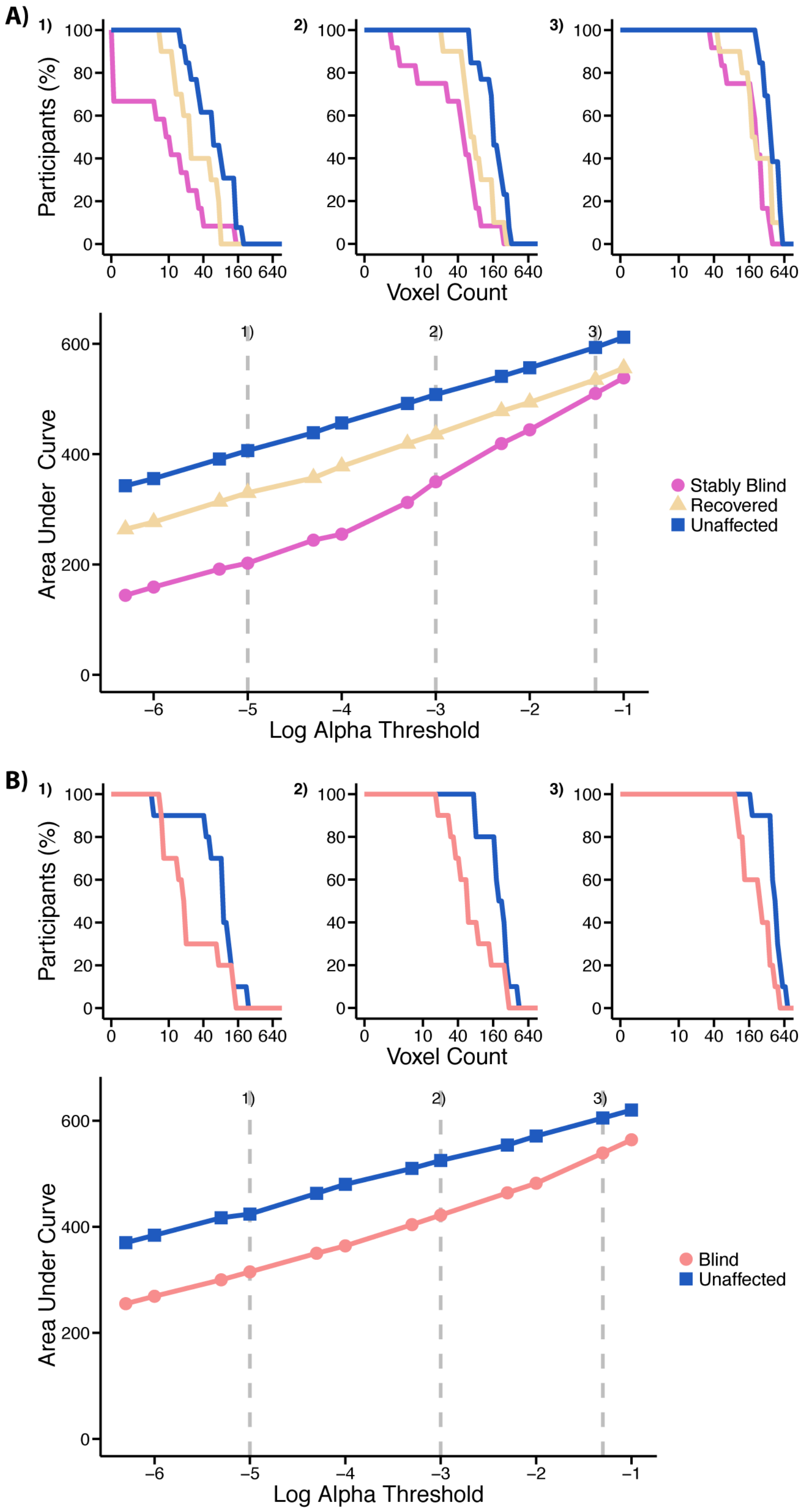
A)
1)
2)
3)
Participants (%)
100
80
60
40
20
0
0
10
40
160
640
Voxel Count
Area Under Curve
600
400
200
0
−6
−5
−4
−3
−2
−1
Log Alpha Threshold
Stably Blind
Recovered
Unaffected
B)
Blind
Unaffected

Figure S4: Variance in final voxel counts across participants between the intact and lesioned hemispheres. Comparisons of the number of significantly active voxels in each stably blind wedge in the lesioned hemisphere (black) versus the mirror image of each stably blind wedge (white) in the intact hemisphere. For example, if the wedge at 2 o'clock was stably blind then the voxel count at 2 o'clock in the lesioned left hemisphere would be normalized with respect to the voxel count at 10 o'clock in the unaffected right hemisphere. A) Average voxel counts, mirror image wedges connected with a dotted line. B) Average of the ratio between the number of active voxels for each stably blind wedge and its mirror image in the intact hemisphere; individual dots represent one mirror-image wedge-pair. C) Average of the log voxel counts; mirror image wedges connected with a dotted line. D) Average of the ratio between the log of the number of active voxels for each stably blind wedge and its mirror image in the intact hemisphere, individual dots represent one mirror-image wedge-pair. Participants 2 and 10 excluded because they had bilateral lesions. Participant 4 did not have fMRI data.

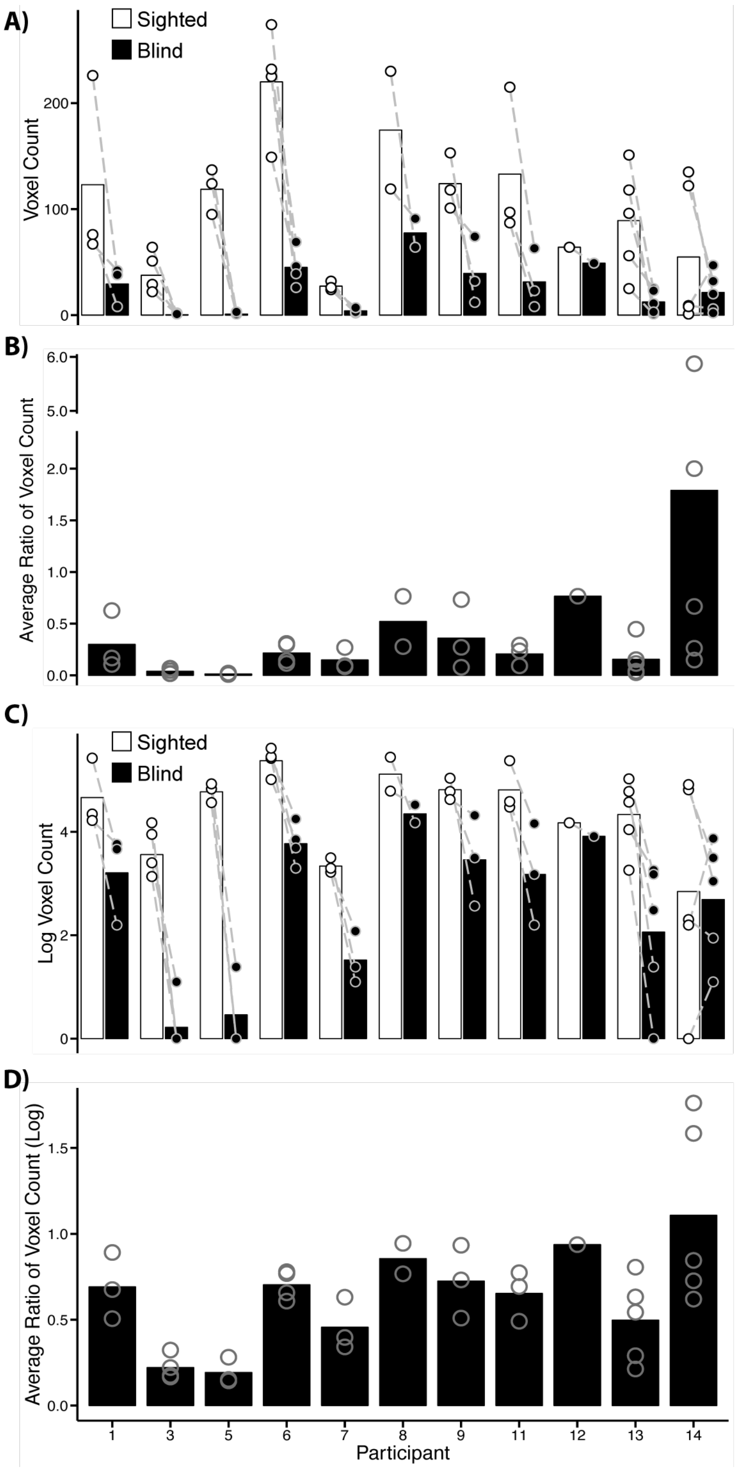

A)
Sighted
Blind
Voxel Count
B)
Average Ratio of Voxel Count
C)
Log Voxel Count
D)
Average Ratio of Voxel Count (Log)
Participant

317

Figure S5: Logarithmic relation between visual cortex activity and GCC thickness at the final time point for stably blind areas of the visual field.

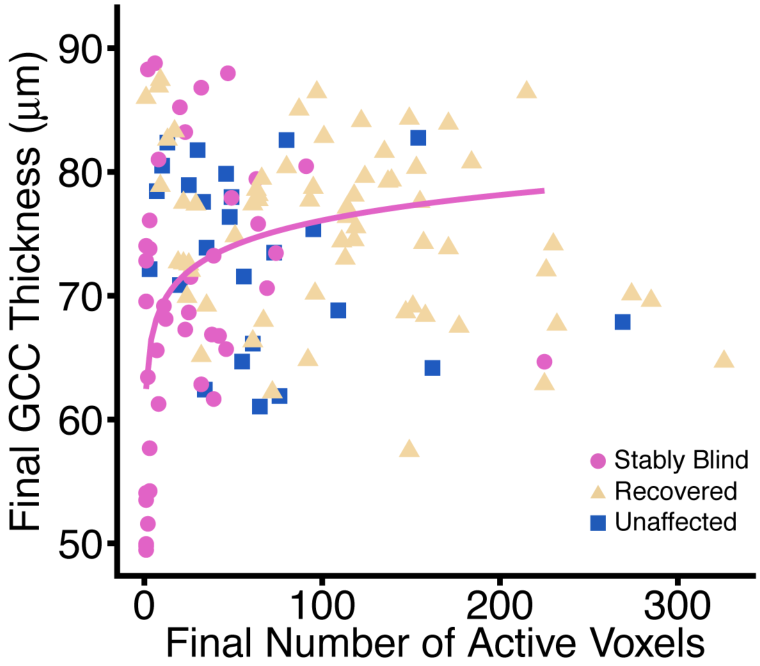

Figure S6: GCC thickness of regions of the retina corresponding to the stably blind field are related to total lesion size and the size of the lesion within the medial occipital lobe. Total lesion area (A) was subdivided into the component that was outside (B) versus inside (C) the early visual cortex medial occipital lobe mask used for all core analyses and correlated with GCC thickness, controlling for time since stroke. Y-axis is residuals of GCC thickness when controlling for time since stroke; n = 15; pink circle – stably blind; yellow triangle – recovered; blue square – unaffected.

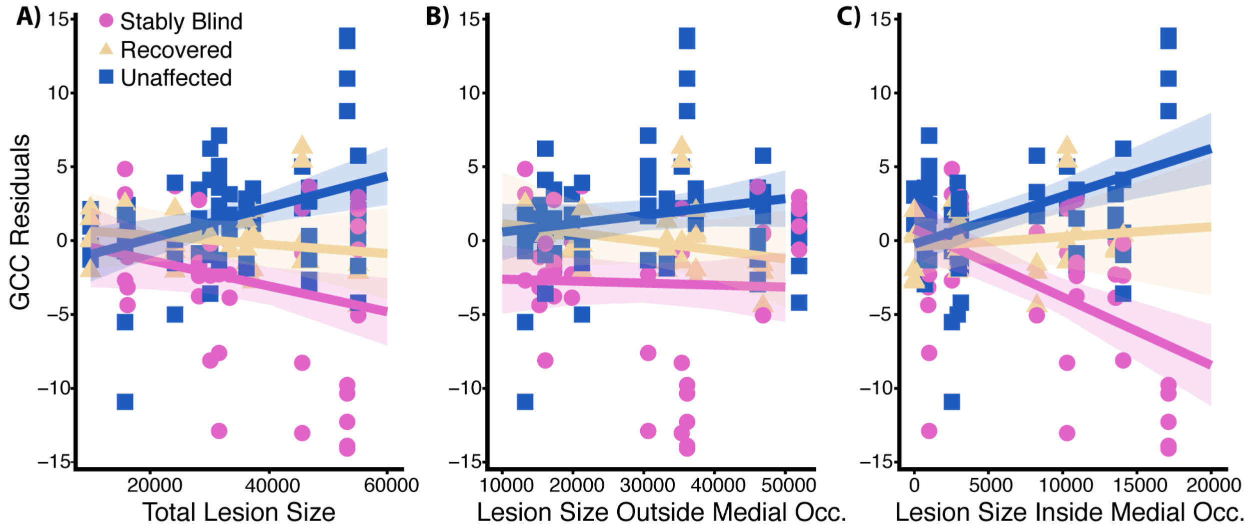

Figure S7: GCC thickness is associated with visual cortex activity for a range of statistical thresholds for defining significantly active voxels. GCC thickness (controlling for time since stroke and total lesion size) as a function of visual cortex activity and visual ability at varying statistical thresholds (1: $p < 0.00001$; 2: $p < 0.001$; 3: $p < 0.05$). Lower panel shows slope of the fits in the top panel as a function of change in vision and statistical threshold. For ease of viewing, alphas are plotted on a log scale (i.e. alpha = 0.001 is equal to $1x10^{-3}$ and is therefore plotted on the x-axis at -3). A) Final fMRI binned by change in vision (n = 13), and B) initial fMRI binned by initial vision (n = 10). Pink circle – stably blind; yellow triangle – recovered; blue square – unaffected; red circle – blind at first time point.

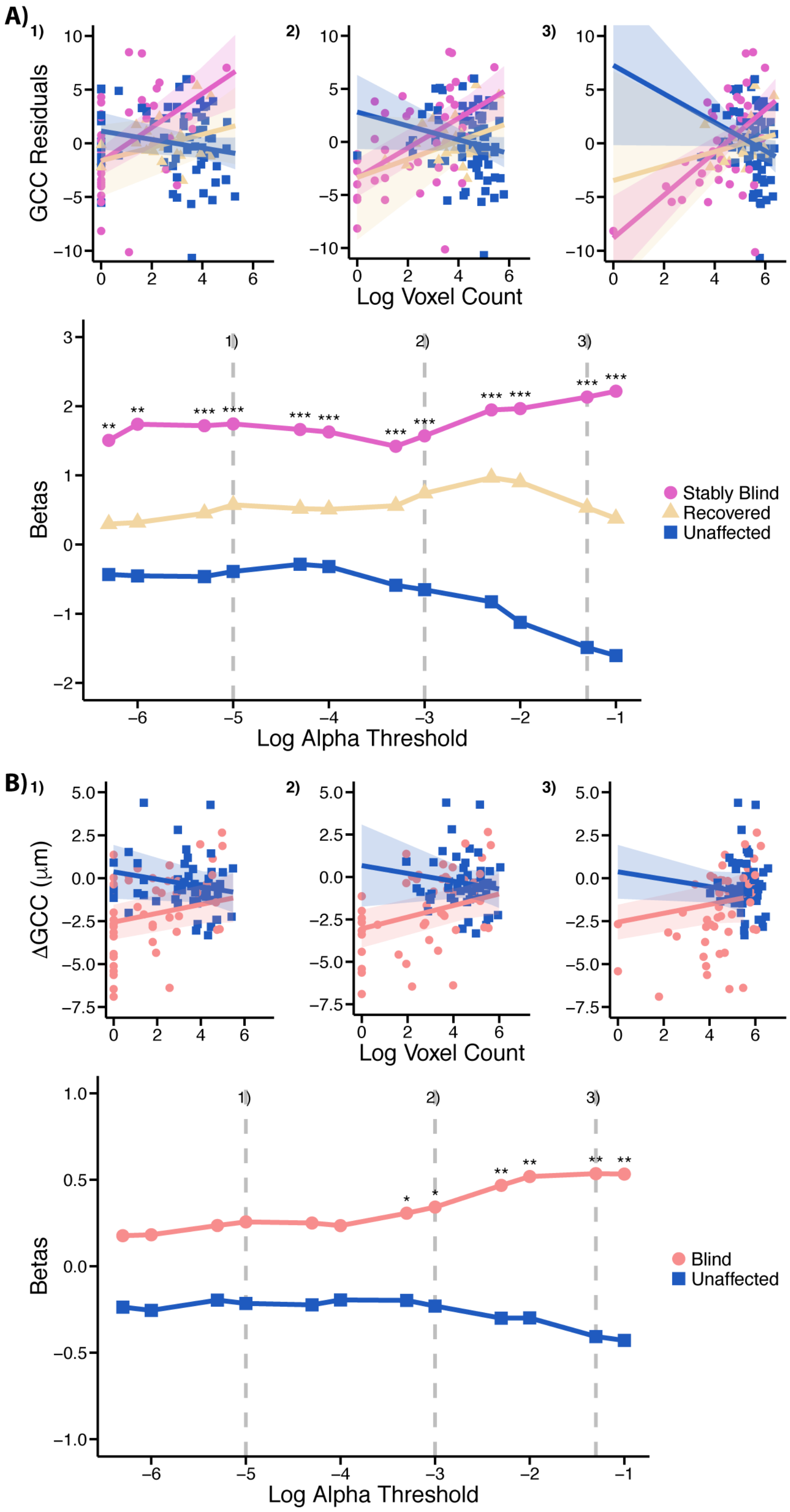
A)
1)
2)
3)
GCC Residuals
Log Voxel Count
Betas
Log Alpha Threshold
Stably Blind
Recovered
Unaffected
B)
ΔGCC (μm)
Log Voxel Count
Betas
Log Alpha Threshold
Blind
Unaffected

Figure S8: Comparison of scanners. Two approaches were pursued to evaluate whether each scanner had sufficient signal-to-noise to support the core measures of neural function. A) temporal signal-to-noise ratios (TSNR) were calculated from two polar angle runs. Maps show the medial surface of four healthy control subjects scanned on three different 3T MRIs. Areas in blue/white have sufficient TSNR (TSNR > 40) to detect statistically significant differences in the BOLD signal between two or more conditions [30,31]. Critically, all scanners have sufficient TSNR in our region of interest (medial occipital lobe). B) We then sought to evaluate whether there is equivalent reproducibility of retinotopic preferences across scanners. This was possible because all of the control/healthy participants completed 2 runs of polar angle mapping on each scanner. We used multivoxel pattern correlation over medial occipital cortex to compare the similarity of the same condition (wedge location) across the two runs (within participant) on the same scanner versus between two runs (again within the same participant) on two different scanners (or for the Trio-Trio comparison, the same scanner for two different sessions). In addition to computing similarity for each condition to itself (within and between scanners) we also computed the average between condition (dis)similarity between runs (again, always within participants). This analysis amounts to comparing the diagonal to off-diagonal values of a representational similarity matrix of all 12 wedge locations to all 12 wedge locations (always between runs, within participants, and either within or between scanners). White bars show the average of the within-condition correlations for all 12 wedges (wedge x > baseline) for two different runs (diagonal of the representational similarity matrix comparing contrast-weighted t-values for two different runs). Black bars show the average of the between-condition correlations among all 12 wedges (wedge x > baseline) for two different runs (off-diagonal of the representational similarity matrix). The average for individual participants are shown by the

dots. All scanner-run combinations show significantly greater within-condition correlations than between-condition correlations. C) Data from B expanded to show performance of individual scanner/session combinations. Importantly, all scanner/session combinations show significantly greater within-condition correlations than between-condition correlations, suggesting that even polar angle maps constructed from data on the same participant but on different scanners have sufficient sensitivity to reliably measure retinotopic preferences.

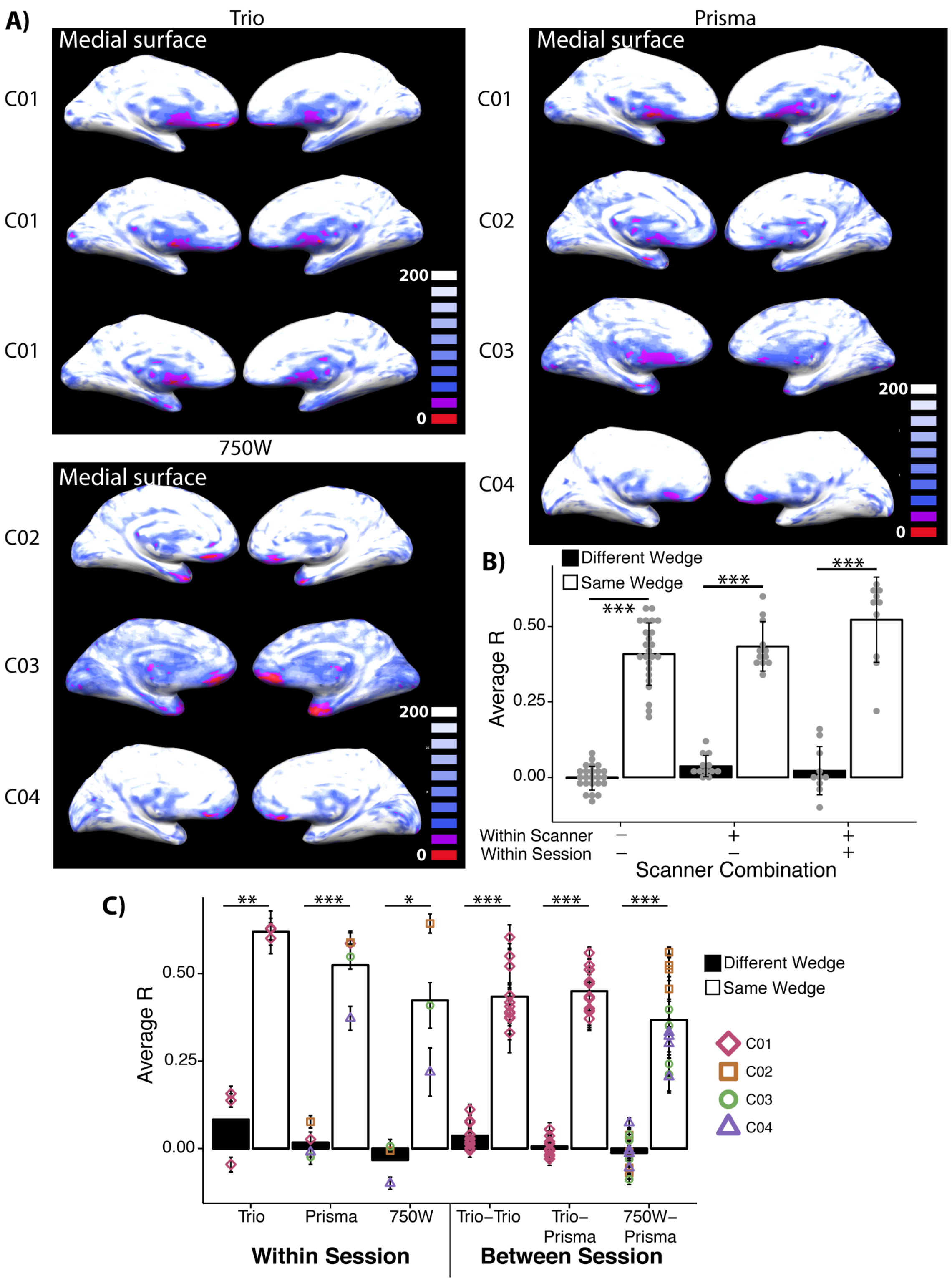
A)
Trio
Medial surface
C01
C01
C01
200
0
Prisma
Medial surface
C01
C02
C03
C04
200
0
750W
Medial surface
C02
C03
C04
200
0
B)
Different Wedge
Same Wedge
***
***
***
Average R
0.50
0.25
0.00
Within Scanner − + +
Within Session − − +
Scanner Combination
C)
** *** * *** *** ***
Average R
0.50
0.25
0.00
Different Wedge
Same Wedge
C01
C02
C03
C04
Trio
Prisma
750W
Trio−Trio
Trio−Prisma
750W−Prisma
Within Session
Between Session

370

371

Figure S9: Cumulative distribution of Humphrey visual field total deviation values. Total deviation values for all participants at all visual field test locations and time points. The boundary for classifying a test location as blind or sighted was set at a total deviation of -6 dB since this is the elbow of the cumulative distribution plot.

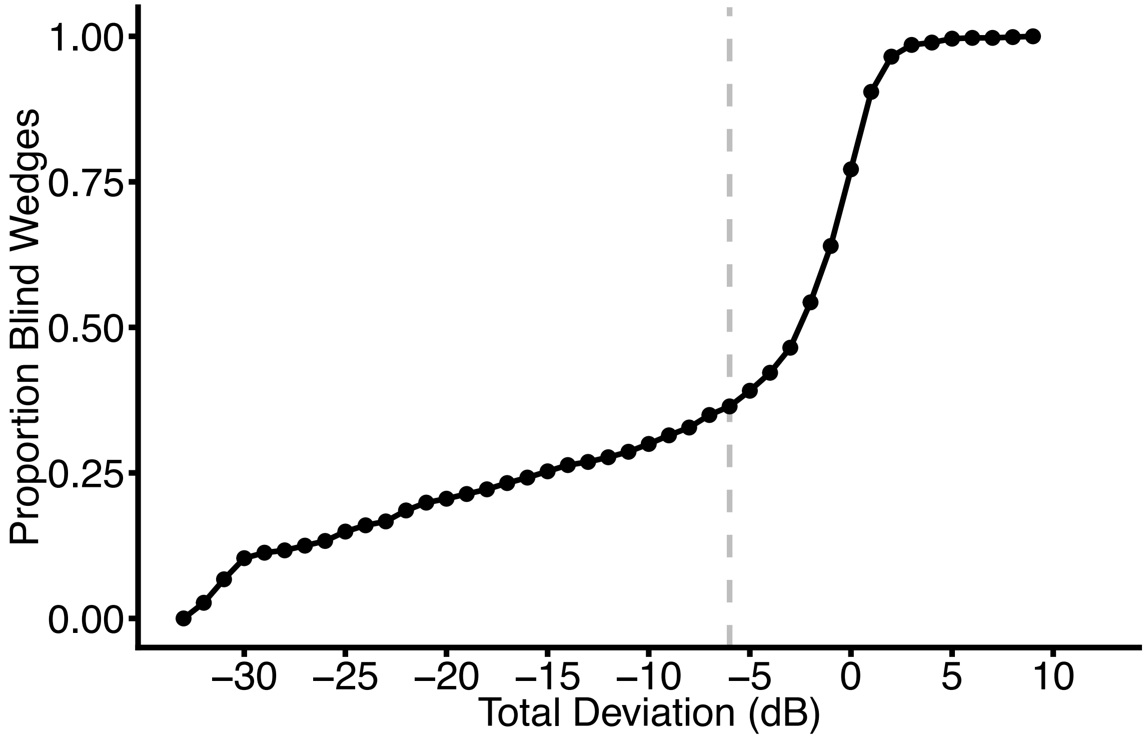

Figure S10: Letter detection and identification task. A) Black letter stimuli were presented on a mean luminance background one at a time in 72 different locations within the central 22.5 degrees of vision. B) Example results from participant 5.

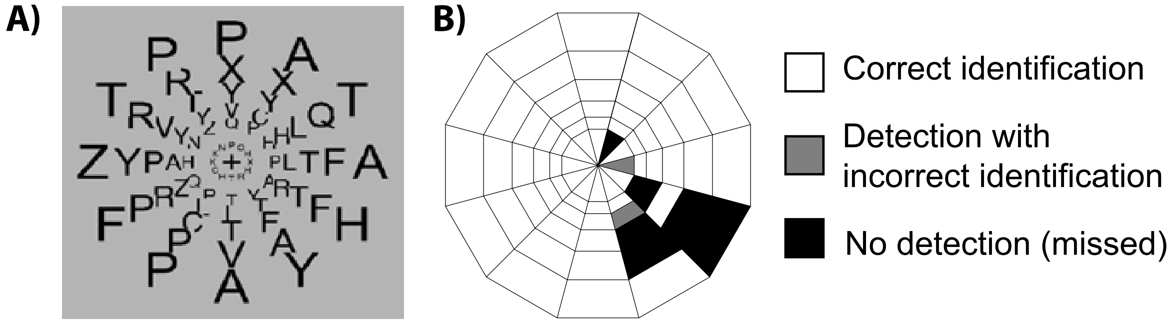

Table S1: Experimental metadata. Standard of care and study visit testing time points for Humphrey visual field testing, OCT, and fMRI retinotopy. All participants completed at least one study visit ≥ 5 months post-stroke; participants 6 – 15 also completed a study visit that included fMRI < 2 months post-stroke. Some participants also completed additional study visits between the first and last visit, which are not reported here or in this manuscript.

	Days Post Stroke					
**Participant**	**Humphrey**	**OCT**	**fMRI**	**Scanner**	**Stimulus**	**Notes**
1	63					Standard of care
	602	602	602	Trim Trio	Random	fMRI: Eye tracking, 2 runs
2	6					Standard of care
	528	528	527	Trim Trio	Sequential	fMRI: Eye tracking, 2 runs
3	4					Standard of care
	675	675	675	Trim Trio	Random	fMRI: Eye tracking, 2 runs
4	5					Standard of care
	324	324				Study visit, no fMRI
5	2		3	750W	Continuous	fMRI data not used due to technical difficulties
	199	199	199	Trim Trio	Random	fMRI: Eye tracking, 2 runs
6	3	3	5	750W	Sequential	fMRI: No eye tracking, 2 runs
	192	192	192	Trim Trio	Random	fMRI: Eye tracking, 2 runs
7	25	25	25	Trim Trio	Random	fMRI: Eye tracking, 2 runs
	297	297	297	Prisma	Random	fMRI: Eye tracking, 2 runs
8	8	8	3	750W	Random	fMRI: No eye tracking, 2 runs
	276	276	276	Prisma	Random	fMRI: Eye tracking, 2 runs
9	63	63	54	Trim Trio	Random	fMRI: Eye tracking, 2 runs
	300	317	300	Prisma	Random	fMRI: Eye tracking, 2 runs
10	5	5	5	Trim Trio	Random	fMRI: Eye tracking, 2 runs
	184	184	184	Prisma	Random	fMRI: Eye tracking, 1 run, other run excluded - participant asleep
11	3	3	3	750W	Sequential	fMRI: No eye tracking, 2 runs
	183	183	183	Trim Trio	Random	fMRI: Eye tracking, 2 runs
12	2	2	1	750W	Random	fMRI: Eye tracking, 2 runs
	261	261	261	Trim Trio	Random	fMRI: Eye tracking, 2 runs
13	3	3	34	Trim Trio	Random	fMRI: Eye tracking, 2 runs
	174	174	174	Trim Trio	Random	fMRI: Eye tracking, 1 run, other run excluded - participant asleep
14	5	13	33	Trim Trio	Random	fMRI: Eye tracking, 2 runs
	270	270	270	Prisma	Random	fMRI: Eye tracking, 2 runs
15	5	5	6	Trim Trio	Random	fMRI: Eye tracking, 1 run, other run excluded - participant asleep
	180	180	180	Prisma	Random	fMRI: Eye tracking, 2 runs, data not used - participant asleep

Table S2: Demographics of healthy control participants.

Participant	Age	Gender	Scanners
C01	27	F	Trio, Prisma
C02	29	F	Prisma, 750W
C03	21	F	Prisma, 750W
C04	27	M	Prisma, 750W

Table S3: Left and right Talairach coordinates used to define medial occipital cortex in each participant.

Participant	Left TalX	Right TalX
1	-19	19
2	-19	13
3	-19	19
4	No fMRI	
5	-25	19
6	-16	16
7	-22	19
8	-19	19
9	-22	19
10	-19	16
11	-22	22
12	-25	19
13	-19	25
14	-16	22
15	-19	19

References

1. Jindahra P, Petrie A, Plant GT. 2009 Retrograde trans-synaptic retinal ganglion cell loss identified by optical coherence tomography. *Brain* **132**, 628–634.

2. Jindahra P, Petrie A, Plant GT. 2012 The time course of retrograde trans-synaptic degeneration following occipital lobe damage in humans. *Brain* **135**, 534–541.

3. Park HL, Park YG, Cho A, Park CK. 2013 Transneuronal retrograde degeneration of the retinal ganglion cells in patients with cerebral infarction. *Ophthalmology* **120**, 1292–1299.

4. Gunes A, Erkol E, Seden I, Levent D, Ozlem T. 2016 Changes in Retinal Nerve Fiber Layer Thickness in Patients with Cerebral Infarction: Evidence of Transneuronal Retrograde Degeneration. *Acta Neurol. Belg.* **116**, 461–466.

5. Keller J, Sanchez-Dalmau B, Villoslada P. 2014 Lesions in the Posterior Visual Pathway Promote Trans-Synaptic Degeneration of Retinal Ganglion Cells. *PLoS One* **9**, 1–5.

6. Schwartz SG, Monroig A, Flynn HW. 2017 Progression of Transsynaptic Retinal Degeneration with Spectral-Domain Optical Coherence Tomography. *Am. J. Ophthalmol. Case Reports* **5**, 67–72.

7. Yamashita T, Miki A. 2012 Reduced retinal ganglion cell complex thickness in patients with posterior cerebral artery infarction detected using spectral-domain optical coherence tomography. *J. Ophthalmol.* **56**, 502–510.

8. Shin H, Park HL, Choi J, Park CK. 2014 Macular ganglion cell – inner plexiform layer thinning in patients with visual field defect that respects the vertical meridian. *Arch. Clin. Exp. Ophthalmol.* **252**, 1501–1507.

9. Cowey A, Alexander I, Stoerig P. 2011 Transneuronal retrograde degeneration of retinal ganglion cells and optic tract in hemianopic monkeys and humans. *Brain* **134**, 2149–2157.

10. Dilks DD, Serences JT, Rosenau BJ, Yantis S, McCloskey M. 2007 Human Adult Cortical Reorganization and Consequent Visual Distortion. *J. Neurosci.* **27**, 9585–9594.

11. Chen Q, Garcea FE, Mahon BZ. 2016 The Representation of Object-Directed Action and Function Knowledge in the Human Brain. *Cereb. Cortex* **26**, 1609–1618.

12. Paul DA, Gaffin-Cahn E, Hintz EB, Adeclat GJ, Zhu T, Williams ZR, Vates GE, Mahon BZ. 2014 White Matter Changes Linked to Visual Recovery after Nerve Decompression. *Sci. Transl. Med.* **6**, 266ra173.

13. Garcea FE, Mahon BZ. 2014 Parcellation of left parietal tool representations by functional connectivity. *Neuropsychologia* **60**, 131–143.

14. Talairach J, Rournoux P. 1988 *Co-planar stereotaxic atlas of the human brain. 3-Dimensional proportional system: an approach to cerebral imaging*. Stuttgart: Thieme.

15. Reitsma DC, Mathis J, Ulmer JL, Mueller W, Maciejewski MJ, DeYoe EA. 2013 Atypical retinotopic organization of visual cortex in patients with central brain damage: congenital and adult onset. *J. Neurosci.* **33**, 13010–13024.

16. de Haan B, Clas P, Juenger H, Wilke M, Karnath H-O. 2015 Fast semi-automated lesion demarcation in stroke. *Neuroimage* **9**, 69–74.

17. Bates E, Wilson SM, Saygin AP, Dick F, Sereno MI, Knight RT, Dronkers NF. 2003 Voxel-based lesion-symptom mapping. *Nat. Neurosci.* **6**, 448–450.

18. Prentiss EK, Schneider CL, Williams ZR, Sahin B, Mahon BZ. 2018 Spontaneous in-flight accommodation of hand orientation to unseen grasp targets : A case of action

blindsight. *Cogn. Neuropsychol.* , 1–9.

19. Colenbrander A. 1999 *Guide for the Evaluation of Visual Impairment*. San Francisco: Pacific Vision Foundation.

20. Tiel K, Kolmel HW. 1990 Patterns of Recovery from Homonymous Hemianopia Subsequent to Infarction in the Distribution of the Posterior Cerebral Artery. *Neuro-Ophthalmology* **11**, 33–39.

21. Wessinger CM, Fendrich R, Gazzaniga MS. 1999 Variability of residual vision in hemianopic subjects. *Restor. Neurol. Neurosci.* **15**, 243–253.

22. Fendrich R, Wessinger CM, Gazzaniga MS. 1992 Residual Vision in a Scotoma: Implications for Blindsight. *Science (80-.).* **258**, 1489–1491.

23. Kentridge RW, Heywood CA. 1992 Residual Vision in Multiple Retinal Locations within a Scotoma: Implications for Blindsight. *J. Cogn. Neurosci.* **9**, 191–202.

24. Schärli H, Harman AM, Hogben JH. 1999 Blindsight in Subjects with Homonymous Visual Field Defects. *J. Cogn. Neurosci.* **11**, 52–66.

25. Schoenfeld MA, Noesselt T, Poggel D, Tempelmann C, Hopf JM, Woldorff MG, Heinze HJ, Hillyard SA. 2002 Analysis of pathways mediating preserved vision after striate cortex lesions. *Ann. Neurol.* **52**, 814–824.

26. Kavcic V, Triplett RL, Das A, Martin T, Huxlin KR. 2015 Role of inter-hemispheric transfer in generating visual evoked potentials in V1-damaged brain hemispheres. *Neuropsychologia* **68**, 82–93.

27. Henriksson L, Raninen A, Nasanen R, Hyvarinen L, Vanni S. 2007 Training-Induced Cortical Representation of a Hemianopic Hemifield. *J. Neurol. Neurosurg. Psychiatry* **78**,

74–81.

28. Klein I, Paradis A-L, Poline J-B, Kosslyn SM, Le Bihan D. 2000 Transient Activity in the Human Calcarine Cortex During Visual-Mental Imagery: An Event-Related fMRI Study. *J. Cogn. Neurosci.* **12**, 15–23.

29. Sperandio I, Chouinard PA, Goodale MA. 2012 Retinotopic activity in V1 reflects the perceived and not the retinal size of an afterimage. *Nat. Neurosci.* **15**, 540–542. (doi:10.1038/nn.3069)

30. Murphy K, Bodurka J, Bandettini PA. 2007 How long to scan? The relationship between fMRI temporal signal to noise ratio and necessary scan duration. *Neuroimage* **34**, 565–574.

31. Anzellotti S, Mahon BZ, Schwarzbach J, Caramazza A. 2011 Differential activity for animals and manipulable objects in the anterior temporal lobes. *J. Cogn. Neurosci.* **23**, 2059–2067.
